# Seroprevalence and Molecular Evidence of *Coxiella burnetii* in Dromedary Camels of Pakistan

**DOI:** 10.3389/fvets.2022.908479

**Published:** 2022-06-16

**Authors:** Shujaat Hussain, Muhammad Saqib, Hosny El-Adawy, Muhammad Hammad Hussain, Tariq Jamil, Muhammad Sohail Sajid, Mughees Aizaz Alvi, Muzafar Ghafoor, Muhammad Haleem Tayyab, Zaeem Abbas, Katja Mertens-Scholz, Heinrich Neubauer, Iahtasham Khan, Muhammad Khalid Mansoor, Ghulam Muhammad

**Affiliations:** ^1^Department of Clinical Medicine and Surgery, Faculty of Veterinary Science, University of Agriculture, Faisalabad, Pakistan; ^2^Institute of Bacterial Infections and Zoonoses, Friedrich-Loeffler-Institut, Jena, Germany; ^3^Faculty Medicine of Veterinary, Kafrelsheikh University, Kafr El-Sheikh, Egypt; ^4^Faculty of Veterinary and Animal Sciences, The Islamia University of Bahawalpur, Bahawalpur, Pakistan; ^5^Department of Parasitology, Faculty of Veterinary Science, University of Agriculture, Faisalabad, Pakistan; ^6^Department of Clinical Sciences, University of Veterinary & Animal Sciences, Lahore Sub Campus Jhang, Lahore, Pakistan

**Keywords:** *Coxiella burnetii*, dromedary camels, zoonosis, ELISA, PCR, risk factors, Pakistan

## Abstract

Coxiellosis is a zoonosis in animals caused by *Coxiella burnetii*. A cross-sectional study was conducted on 920 (591 female and 329 male) randomly selected camels (*Camelus dromedarius*) of different age groups from 13 districts representative of the three different ecological zones in the Province Punjab, Pakistan to determine the prevalence and associated risk factors of coxiellosis. The blood samples were collected and tested for anti-*C. burnetti* antibodies using indirect multispecies ELISA. Real-time PCR was used for the detection of *C. burnetii D*NA to determine the prevalence in heparinized blood pools. Out of 920 investigated camels, anti-C. *burnetii* antibodies were detected in 288 samples (31.3%) (95% CI: 28.3–34.4%). The highest (78.6%) and lowest (1.8%) seroprevalence were detected in Rahimyar Khan (southern Punjab) and in Jhang (central Punjab), respectively. Potential risk factors associated with seropositivity of the Q fever in camels included desert area (42.5%; OR = 2.78, 95% CI 1.12–3.21) summer season (35.7%; OR = 2.3, 95% CI: 1.31–3.2), sex (female) (39.1; OR = 2.35, 95% CI: 1.34–2.98), tick infestation (51.3%;OR = 2.81, 95% CI: 1.34–3.02), age (>10 years; 46.4%; OR = 1.56, 95% CI: 0.33–2.05) and herd size (38.5%; OR = 1.21, 95% CI: 0.76–1.54). *Coxiella burnetii* DNA was amplified in 12 (20%) and 1 (10%) of 60 ELISA-negative and 10 suspected camels, respectively. DNA could not be detected in ELISA positive blood pools. This study emphasizes the seroprevalence and associated risk factors of coxiellosis as well as its potential to spill over to animals and humans in contact with these camel herds.

## Introduction

Coxiellosis (Q fever) is associated with ticks and is a neglected zoonosis at least in the developing countries caused by the intracellular γ-proteobacterial pathogen, *Coxiella* (*C*.) *burnetii* (1, 2).

*Coxiella*-like bacteria and *C. burnetii* are closely related, they vary in their ecology, as illustrated by the differences observed in transmission routes and infectiousness. Recent investigations based on multilocus phylogenetic analyses and whole-genome sequencing data revealed that all known *C. burnetii* strains originated within the vast group of *Coxiella*-like endosymbionts and are the descendants of a *Coxiella*-like progenitor hosted by ticks (3). In this context, comparative genomic approaches will be highly valuable in enhancing understanding of the evolutionary ecology of both *C. burnetii* and *Coxiella*-like bacteria and in identifying genes involved in virulence and tick symbiosis.

Based on the structural variation in lipopolysaccharide (LP), two antigenic “phases” of the organism viz., phase I (virulent) and phase II (avirulent), exist (4). *C. burnetii* infection has been reported in humans, animals (both wild and domestic), and ticks (1, 5, 6). Domestic ruminants (sheep and goat) act as reservoirs for *Coxiella* and are usually incriminated as an origin of Q fever epidemics in humans (2, 7). In humans, the symptoms of Q fever are non-specific; however, the acute disease manifests with fever, myalgia, and atypical pneumonia. The chronic infection develops following an acute course and may lead to endocarditis and vasculitis (8–10).

Livestock farmers, shepherds, veterinarians, abattoir workers, and laboratory technicians have a higher risk of *C. burnetii* infection (11, 12). Q fever is often asymptomatic in the livestock; however, late abortion, stillbirth, and premature delivery can be seen in small ruminants (sheep and goats) whilst mastitis, metritis, and infertility are reportedly observed in cows (13). In ruminants, *C. burnetii* is shed in birth fluids, uterine discharge, placental tissues, milk, urine, feces, and semen. Although inhalation of *C. burnetii* is considered the major route of infection in humans; however, consumption of raw milk and milk byproducts, blood transfusion, transplacental infection, intradermal inoculation, and infection after contact with the infected animals' body secretions *viz*, urine, feces, and semen may occur (9). The involvement of sheep and goats in human outbreaks of Q fever is well-documented (14). However, the high prevalence of coxiellosis found in camels, raise the question of whether transmission of *C. burnetii* from camels to humans is possible (15).

Numerous sero-surveys of coxiellosis in camels have been conducted and reported as 66% positive in Egypt (16), 80% in Chad (17), 62% in Saudi Arabia (18), 71.2% in Algeria (19), 44% in Tunisia (20), 29% in Iran (21) and nearly 100 % in nomadic camels in southeastern Ethiopia (22). The studies published over the last 6 decades (23–28) display that Q fever has been a neglected zoonosis in Pakistan. To our best, serologic evidence of Q fever in Pakistani camels can be traced back to 1955 (23) but no epidemiologic data on the disease in camels was available later.

A variety of diagnostic tests are available for the diagnosis of *C. burnetii* infection in animals. Nevertheless, ELISA and PCR are believed to be among the most reliable methods for serological and molecular diagnosis, respectively (29). In view of the emergence of coxiellosis in camels and the scarcity of epidemiologic data in Pakistan, this study was designed to investigate sero-prevalence, and associated risk factors among one-humped (dromedary) camels (*Camelus dromedarius*) reared in Pakistan.

## Materials and Methods

### Study Area

A cross-sectional sero-epidemiological survey was conducted from June 2018 to December 2019 in various districts of Punjab, Pakistan. According to Livestock Census, 13 camels were kept in prefectures from central, southern, and north-western parts of Punjab. Geographic coordinates, climatic conditions, and camel population of the selected districts are shown in Table 1.

**Table 1:** Coordinates and climatic characteristics of the study districts of Punjab province, Pakistan.

**Punjab province (Zone)**	**Districts**	**Coordinates**		**Elevation above sea level (ft)**	**Average temperature (** **°** **C)**				**Camel Population**
		**Latitude (**°**N)**	**Longitude (**°**E)**		**Summer**		**Winter**		
					Max.^*^	Min.^*^	Max.^*^	Min.^*^	
Central	Faisalabad	31° 27'	73° 8'	607	39	27	21	6	687
	Chiniot	31° 43'	72° 58'	597	40	27	19	6	663
	Jhang	31° 16'	72° 19'	515	41	28	19	6	1,265
Northern	Mianwali	32° 35'	71° 32'	705	40	26	19	5	1,886
	Bhakkar	31° 37'	71° 3'	561	41	28	19	6	5,310
	Khushab	32° 18'	72° 17'	600	40	27	19	5	3,712
	Sargodha	32° 5'	72° 40'	620	40	27	19	5	774
Southern	Bahawalnagar	30° 0'	73° 15'	511	41	28	20	4	681
	Layyah	25° 20'	55° 22'	479	41	29	19	7	3,155
	Muzaffar Garh	32° 7'	80° 3'	390	42	28	20	7	1,687
	Bahawalpur	29° 21'	71° 41'	370	41	29	19	6	1,078
	Rahim Yar Khan	28° 35'	77° 14'	272	41	28	23	5	1,921
	Lodhran	29° 32'	71° 37'	377	41	28	20	7	115

* *Max., Maximum; Min., Minimum*.

### Sampling Frame

The sample size was calculated by considering the expected disease prevalence up to 50% with a confidence interval (CI) of 99% and a desired absolute precision of 5%. The sample size was further expanded to improve the degree of accuracy and to cover the expected losses during handling and transportation from remote areas. A minimum of 897 samples were calculated to be needed for this survey. In practice, a total of 920 camels (591 females and 329 males) were randomly sampled from 13 districts of Punjab, Pakistan. Blood samples were drawn into a 4 mL, gel-clot activator and EDTA coated vacuum vials, separately. The sera were harvested upon centrifugation and preserved (-40°C) for further investigations. Meta data (age, breed, sex, body score, tick infestation, reproductive history/problems, and location, etc.) along with season and management/herd type were recorded on a questionnaire at the time of sampling. The animals were categorized into 3 different age groups; ≤ 5 years (*n* = 269), >5 to 10 years (*n* = 348) and >10 years of age (*n* = 303). For a random selection of herds and animals survey toolbox software was used (30).

### Ethics' Statement

Blood samples were collected from camels as per the guidelines of the International Animal Care and Use Committee (IACUC) and after obtaining written consent from the owner of the camel. The study was approved by the Directorate of Graduate Studies following the ethical guidelines of the Institutional Biosafety Committee (IBC) and the Institutional Animal Care and Use Guidelines given in the Animal Care Handbook of the University of Agriculture, Faisalabad, Pakistan (31).

### Serological Testing

Detection of *anti-C. burnetii* (against phase I and II antigens) antibodies were carried out by the commercially available indirect ELISA kit for the Q fever (ID Screen^®^ Q fever indirect Multi-species ELISA, IDvet, Grabels, France) following the manufacturer's recommendations, and the results were expressed as optical density (OD) values. The absorbance was measured by an ELISA plate reader (Multiscan FC, Thermofisher Scientific, USA) at 450 nm. Sample/positive percentages (S/P%) for individual serum samples were calculated by using the following formula:

[(OD sample–OD negative)/(ODpositive–ODnegative)] ×100. Samples were considered negative if they had S/P % ≤ 40%, doubtful for values between 40 and 50% and positive for S/P % > 50%. Any serum sample that was initially classified as “doubtful” was retested.

### Molecular Investigation

Molecular testing was carried out on the blood pools (each pool comprised of five blood samples) from sero-negative (*n* = 60), positive (*n* = 11) and suspected (*n* = 10) camels using commercially available TaqMan-based real-time PCR assay (32). The DNA was eluted using a genomic DNA extraction kit (GeneJET Genomic DNA Purification Kit, Thermo Fisher, Germany), following the manufacturer's instructions, and quantified by Nanodrop 2000 spectrophotometer (Thermofisher Scientific, Germany) and stored at −40°C until used.

Extracted DNA was tested for *C. burnetii* DNA using real-time PCR kit (Liferiver ^TM^ Shanghai ZJ Bio-Tech Co., Ltd.) which is based on the fluorogenic 5′ nuclease assay according to the manufacturer's recommendations. The PCR reaction was performed on BIO-RAD CFX96^TM^ Real Time System (BIO-RAD Laboratories, Inc. USA) with the following protocol; First cycle at 37°C for 2 min, second cycle at 94°C for 2 min, followed by 40 cycles at 93°C for 15 s and at 60°C for 1 min. A sample was considered positive if the value of the threshold cycle (Ct) of the target gene was ≤38 (33). Both negative and positive controls were run in tandem with the samples (34).

### Statistical Analyses

Univariate and multivariate analyses were conducted to determine the association of the risk factors with the seroprevalence. Variables kept in the initial model: district zone, season, breed, age, sex, body condition score (BCS), herd size, husbandry system, reproductive disorder history, and bioclimatic zones (*p* < 0.2 in the univariable analysis). BCS, herd size, season, breed, and husbandry system were removed in subsequent steps (*p* > 0.05). A *p* ≤ 0.05 was considered as a level of significance. A backward stepwise approach was used for the binary logistic regression analysis (35). All variables with a *p* < 0.2 in the initial bivariable analysis, were used to construct a multivariable analysis. Based upon likelihood ratio tests, variables were removed one by one to construct a logistic regression model. Outliers were identified at the 0.5 cut-off point by observing the Hosmer-Lemeshow test, Nagelkerke R square, and residual statistic values used to assess the model-fitness (36). The statistical analysis was conducted using the IBM SPSS Statistics (IBM Corporation, Armonk, NY, USA). The maps were generated by using ArcGIS (ESRI, Redlands, CA, USA).

## Results

### Seroprevalence of *C. burnetii* and Univariable Analysis

An overall sero-prevalence of 31.3% (288/920; CI 28.3–34.4%) was found in the camels of thirteen districts of Pakistani Punjab. The sero-prevalence values varied significantly (*p* < 0.05) between the districts with the highest values in districts Bahawalnagar 78.6% (44/56; CI 65.6–88.4), Rahim Yar Khan 71.4% (40/56; CI 57.8–82.7), Lodhran 68.6% (35/51; CI 54.1-80.9), and Bahawalpur 66.1% (37/56; CI 52.2–78.2). The sero-prevalence varied significantly (*p* < 0.05) between the zones e.g. Southern Punjab showed 50.3% (190/378; CI 45.1–55.4), Northern Punjab 23.7% (88/371; CI 19.5–28.4), and Central Punjab 5.9% (10/171; CI 2.8–10.5) (Table 2).

**Table 2:** Prevalence of Q fever in camels in different ecological zones of Punjab Pakistan.

**Zone**	**District**	**Pos./Tested**	Prev.% (95% CI)^*^
Central Punjab	Chiniot	4/55	7.3 (2.4–18.42)
	Faisalabad	5/60	8.33 (3.11–19.11)
	Jhang	1/56	1.8 (0.09–10.82)
	**Total**	10 / 171	5.8 % (2.84–10.499
Northern Punjab	Bhakkar	25/133	18.8 (12.75–26.69)
	Khushab	48/126	38.1 (29.72–47.21)
	Mianwali	9/56	16.1 (8.1–28.83)
	Sargodha	6/56	10.71 (4.43–22.6)
	**Total**	88 / 371	23.72 % (19.48–28.38)
Southern Punjab	Bahawalnagar	44/56	78.6(65.2–87.98)
	Bahawalpur	37/56	66.1 (52.09–77.84)
	Layyah	31/103	30.1 (21.66–40.05)
	Lodhran	35/51	68.62 (53.97–80.5)
	Muzaffargarh	3/56	5.4 (1.4–15.81)
	Rahimyar Khan	40/56	71.43(57.59–82.32)
	**Total**	190 / 378	50.3% (45.11–55.42)
Total	**288/920**	**31.3 (28.3–34.4)**

* *Seroprevalence varied significantly among different districts, χ^2^ = 263.862, df = 12, p < 0.001. Bold values represents the total of collected and positive tested samples*.

**Figure 1:**
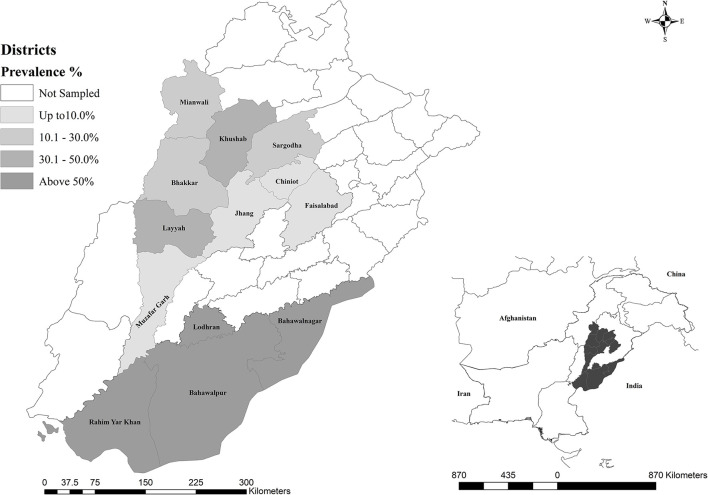
Geographical representation of dromedarian coxiellosis in sampled districts of Punjab, Pakistan.

The geographical distribution of the seroprevalence coxiellosis in sampled districts of Punjab, Pakistan is shown in Figure 1.

Significantly higher seroprevalence (*p* < 0.05) was found in summer 35.7% (221/619; CI 31.9–39.6) compared to winter 22.3% (67/301; CI 17.7–27.4). Sero-prevalence varied significantly (*p* < 0.05) between the breed of the animal where Barella showed higher rates 38.9% (122/314; CI 33.4–44.5) than Marecha 28.6% (108/378; CI 24.1–33.4) and non-descript animals 25.4% (58/228; CI 19.9–31.6). Older animals (>10 years) showed significantly higher (*p* < 0.05) sero-prevalence 46.5% (125/269; CI 40.4–52.6) than younger animals (≤10 years) 25% (163/651; CI 21.8–28.6). Female animals showed significantly higher (*p* < 0.05) sero-prevalence of 39.1% (231/591; CI 35.1–43.2) compared to males 17.3% (57/329; CI 13.4–21.9). Body condition score (BCS), contact with ruminants, and other animals didn't show significant association with the sero-prevalence (*p* > 0.05). Animals with ticks' exposure showed significantly higher (*p* < 0.05) sero-prevalence of 51.3% (140/273; CI 45.2–57.4) compared to animals without exposure 22.9% (148/647; CI 19.7–26.3). Smaller herds (<20 camels) showed significantly higher (*p* < 0.05) seroprevalence of 38.5% (225/585; CI 34.5–42.5) compared to the larger herds (>20 camels) 18.8% (63/335; CI 14.8–23.4). The intensive husbandry system showed a significantly higher (*p* < 0.05) rate of sero-prevalence of 39% (98/251; CI 33.0–45.4) followed by extensive and semi-intensive systems which showed 28.9% (101/349; CI 24.2–34.0) and 27.8% (89/320; CI 23.0–33.1), respectively.

No significant differences were observed for other risk associated factors including ruminant contact and contact with other camel herds. The purpose of the animal e.g. milk, meat, or draft purpose didn't vary significantly in univariable analysis. Presence/history of reproductive disorders in the herd showed significantly higher (*p* < 0.05) sero-prevalence 60.9% (142/233; CI 54.4–67.2) than not present 21.3% (146/687; CI 18.2–24.5). The seroprevalence varied significantly (*p* < 0.05) between the climatic zones where the highest rates were found in desert i.e. 42.5% (225/350; CI 38.2–46.8) whereas plains and arid/semi-arid zones showed almost similar sero-prevalence rates (Table 3).

**Table 3:** Univariable analysis of the potential risk factors of Coxiellosis in One-humped Camels of Punjab, Pakistan.

**Variable**	**Category**	**Pos/Tested**	**Prev (95%CI)**	**OR**	**95%CI**	***p*-Value**
District Zone	Southern	190/378	50.3 (45.1–55.4)	16.27	8.33–31.79	*χ^2^* 124.643 *p* < 0.001
	Northern	88/371	23.7 (19.5–28.4)	5.01	2.53–9.90	
	Central	10/171	5.9 (2.8–10.5)	Ref	-	
Season	Summer	221/619	35.7 (31.9–39.6)	1.94	1.41–2.66	*χ^2^* 17.020 *p* < 0.001
	Winter	67/301	22.3 (17.7–27.4)	Ref	-	
Breed	Barella	122/314	38.9 (33.4–44.5)	1.86	1.28–2.71	*χ^2^* 13.282 *p* = 0.001
	Marecha	108/378	28.6 (24.1–33.4)	1.17	0.81–1.70	
	Non-Descript	58/228	25.4 (19.9–31.6)	Ref	-	
Age	>10 Years	125/269	46.5 (40.4–52.6)	2.60	1.93–3.50	*χ^2^* 40.650 *p* < 0.001
	<10 Years	163/651	25.0 (21.8–28.6)	Ref	2.00–4.33	
Sex	Female	231/591	39.1 (35.1–43.2)	3.06	2.20–4.26	*χ^2^* 46.540 *p* < 0.001
	Male	57/329	17.3 (13.4–21.9)	Ref	-	
BCS	2	07/28	25 (10.7–44.9)	Ref	-	*χ^2^* 2.120 *p* = 0.548
	3	151/452	33.4 (29.1–38)	1.5	0.63–3.62	
	4	128/434	29.5 (25.2–34)	1.25	0.52–3.03	
	5	02/06	33.3 (4.3–77.7)	1.5	0.22–10.04	
Ticks	No	148/647	22.9 (19.7–26.3)	Ref	-	*χ^2^* 72.045 *p* < 0.001
	Yes	140/273	51.3 (45.2–57.4)	3.55	2.63–4.79	
Herd size	Large >20 = 1	63/335	18.8 (14.8–23.4)	Ref	-	*χ^2^* 38.269 *p* < 0.001
	Small <20 = 2	225/585	38.5 (34.5–42.5)	2.73	1.98–3.76	
Husbandry system	Intensive	98/251	39.0 (33.0–45.4)	1.66	1.17–2.36	*χ^2^* 9.68 *p* = 0.007
	Extensive	101/349	28.9 (24.2–34.0)	1.06	0.75–1.48	
	Semi-intensive	89/320	27.8 (23.0–33.1)	Ref	-	
Contact with ruminants	No	129/448	28.8 (24.6–33.2)	Ref	-	*χ^2^* 2.57 *p* = 0.109
	Yes	159/472	33.7 (29.4–38.1)	1.26	0.95–1.66	
Contact with other camel herds	No	150/465	32.3 (28–36.7)	1.09	0.83–1.45	*χ^2^* 0.43 *p* = 0.513
	Yes	138/455	30.3 (26.1–34.8)	Ref	-	
Purpose	Milk	95/300	31.7 (26.4–37.3)	1.03	0.73–1.45	*χ^2^* 0.03 *p* = 0.983
	Meat	99/318	31.1 (26.1–36.5)	1	0.71–1.41	
	Transportation	94/302	31.1 (25.9–36.7)	Ref	-	
Reproductive disorders in herd	No	146/687	21.3 (18.2–24.5)	Ref		*χ^2^* 127.469 *p* < 0.001
	Yes	142/233	60.9 (54.4–67.2)	5.78	4.20–7.97	
Bioclimatic zone	Arid / Semi-Arid	09/56	16.1 (7.6–28.3)	Ref	-	*χ^2^* 72.260 *p* < 0.001
	Desert	225/530	42.5 (38.2–46.8)	3.85	1.85–8.02	
	Plain Area	54/334	16.2 (12.4–20.6)	1.01	0.47–2.18	

### Multivariable Logistic Regression Analysis

Variables with *p* < 0.2 (district zone, season, breed, age, sex, BCS, herd size, husbandry system, reproductive disorder history & bioclimatic zones) were tested by the binary logistic regression model. BCS, herd size, season, breed & husbandry system were removed in subsequent steps (*p* > 0.05). The final model showed that camels: kept in the southern (OR 9.78, CI 1.22–6.16) and northern district zones (OR 2.22, CI 0.94–5.27), >10 years of age (OR 2.52, CI 1.72–3.68), female sex (OR 2.17, CI 1.46–3.21), exposed to ticks (OR 3.39, CI 2.33–4.96), history of reproductive disorders (OR 6.38, CI 4.31–9.44) and desert climate (OR 1.9, CI 1.23–2.95) were found significantly (*p* < 0.05) more likely to test positive (Table 4).

**Table 4:** Potential risk factors influencing the seroprevalence of *Coxiella burnetii* infection among camels in present study (Multivariable analysis).

**Variable**	**Exposure variable**	**Comparison**	**OR**	**95% CI**	***p-Value***
District zone	Southern	Central	9.78	1.22–6.16	<0.001
	Northern	Central	2.22	0.94–5.27	
Age	>10 Years	<10 Years	2.52	1.72–3.68	<0.001
Sex	Female	Male	2.17	1.46–3.21	<0.001
Ticks	Yes	No	3.39	2.33–4.96	<0.001
Reproductive disorder	Yes	No	6.38	4.31–9.44	<0.001
Bioclimatic zone	Desert	Others	1.9	1.23–2.95	0.004

*Model Fit: Nagelkerke R^2^ = 0.326, Hosmer and Lemeshow Test (χ^2^ = 18.553, p = 0.017)*.

### Real-Time PCR

The rate of DNA detection differed significantly among blood pools of camels with different serologic statuses i.e., seronegative, doubtful, and sero-positive. Real-Time PCR demonstrated infection rate in sero-negative and doubtful pools at 20 and 10%, respectively. *C. burnetii* DNA could not be detected in the blood pools of sero-positive (*n* = 11) camels (Table 5).

**Table 5:** Detection of *Coxiella burnetii* DNA in pooled blood samples.

**Category of pools**	**Positive/Tested**	**Prevalence %**	**95% CI**	**Odds ratio**	**95% CI**
Seronegative	12/60	20	22.83–31.78	4.75	
Doubtful	1/10	10	1.79–40.41	2.0	χ^2^ = 46.11 df = 2 *p* = 0.0000
Seropositive	0/11	0	0–25.88	-	-
Overall	**13 /81**	**16.05**	**9.63–25.55**		

*Bold values represents the total of collected and positive tested samples*.

## Discussion

The purpose of the present study was to determine the seroprevalence of anti-*C. burnetii* antibodies and associated risk factors in the dromedary camel population of Punjab, Pakistan. To the best of our knowledge, this is the first comprehensive investigation on coxiellosis in camels of Pakistan. This study on coxiellosis in camels revealed a significantly high prevalence. Out of 920 sampled camels, 288 (31.3%) were seropositive. These findings are similar to those of studies from Iran where seroprevalences varied from 10.7 to 29 % (21, 37). However, this prevalence was lower than that observed in Saudi Arabia (62%) (18), Egypt (66%) (16), and Chad (80%) (17). This might be due to that those camels had the highest off all ruminants (38, 39). The higher prevalences of coxiellosis in camels may be due to genetic susceptibility of camels to *C. burnetii* infection (22) or predilections of tick vectors to camels. Seropositivity differs significantly (*p* < 0.05) among different districts of Punjab, Pakistan, with peak prevalence (78.6%) at Bahawalnagar and the lowest (1.8%) at Jhang, which might be attributed to prevailing climatic conditions, hygienic measures, and management practices. Although, antibodies against coxiellae were detected throughout the year in the present study; however, a peak of detection was observed in Summer (35.7%). This is in close agreement with the previous study conducted by Danish investigators on dairy cows where it was demonstrated that the cows were at a higher risk of *C. burnetti* in summer (40). However, the results of this study are in contrast with those from France, where the human infection is associated with the lambing season in October and November (5).

This study documented that seroprevalence of *C. burnetii* among dromedaries was having a significant positive association with age as the prevalence in aged camels was higher (46.5%) than in younger ones (14.9%). The probable reason might be that the older animals have an extended duration of exposure to the pathogens in the environment causing higher probability of infection than that in the young stock. This observation agrees with previous studies (19, 41) which presented that the seropositivity of *C. burnetii* increases with age. In a similar pattern, seropositivity in the domestic animals (cattle, sheep, and goat) upsurges with the age (22, 42, 43). The same is correct in the case of humans where the prevalence of *C. burnetii* (Q fever) increases with the advancement in age (44, 45).

The sex of the animals was one of the dominant risk factors. Female camels were more often positive (39.1%) when compared to males (17.33%). These results are concomitant with those of previous studies (19, 21). Higher susceptibility of females, particularly aged females, might be due to the predilection of *C. burnetii* to the placenta, udder and other reproductive tissues (46). These tissues can carry up to one billion organisms per gram (47).

The results of the current study revealed a statistically significant association (*p* < 0.05) between tick infestation and the existence of anti-*C. burnetii* antibodies which are in agreement with those reported elsewhere endorsing the vital role of ticks in the maintenance and spread of *C. burnetii* infection among animals and humans (9, 48, 49).

In this study, the herd size was another potentially associated risk factor for seropositivity of *C. burnetii* being higher among dromedary camels belonging to smaller herds (38.5%). This is in contradiction with the precedent studies (19, 21, 43) documenting higher seroprevalence in the larger herds. Our finding of a higher prevalence rate in small-sized herds can be elucidated by the reality that camels belonging to small herds were restricted in close sheds and hence, more prone to inhalation of the infected aerosol (50).

The husbandry system associated significantly with the seropositivity i.e. intensive husbandry system showed higher seropositivity compared to an extensive and semi-intensive system (Table 2). This might be due to a higher risk of contact between the animals e.g., common watering points were found to be a source of brucellosis transmission in camels of Muzaffargarh (51).

The bioclimatic zone is one of the significant risk factors for seropositivity. Seroprevalence was higher in dromedary camels belonging to the desert areas. This finding with those results described in Iran demonstrates a high seroprevalence in the desert and blowy areas (21). Thus, camelids from desert areas are more prone to aerosol transmission due to the frequency of storms in desert areas.

The breed was a risk factor in univariate analysis, which is in close agreement with the previous reports that recognized the breed as a risk factor for coxiellosis in cattle and sheep (40, 49). There is no association between a history of abortion and seropositivity observed in the current study. This finding is in agreement with previous findings in Saudi Arabian (18). Such an association has been discussed repetitively in the literature for other animal species as well (49). Unexpectedly the husbandry system was not statistically significant for seropositivity.

Additional risk factors like ruminant contact, contact with other camel herds, purpose, and reproductive disorders possibly be associated with the seroprevalence of *C. burnetii* were not found significant in the current study. Contribution to other ruminants was not significant in this study. This agrees with previous findings in Algeria (19). In the current work, no significant association was observed with seropositivity. This is in contrast with previous findings from Tunisia which reported high seroprevalence in camels intended for meat production (20). Reproductive disorders potentially associated with seropositivity were not statistically significant in the present study; however, these findings are not consistent with the earlier reports (49, 52–54) describing a higher prevalence associated with the reproductive disorders.

Other factors e.g. body score (BCS), ruminant contact, contact with other camel herds, and purpose were not found significant in the current study. This agrees with previous findings in Algeria (19). This is in contrast with previous findings from Tunisia which reported high seroprevalence in camels intended for meat production (20).

The overall prevalence of *C. burnetii* DNA in the camel blood samples was 16.05 % which is in close agreement with the previous findings in Iran (37). These results indicated that new infections play an important role in camels in Punjab, Pakistan as the agent is no longer present in the blood in which antibodies have been formed.

## Conclusions and Recommendations

The findings of this study indicated that coxiellosis is prevalent in clinical and/or subclinical forms in the camel population of different agro-geo-climatic zones of Punjab, Pakistan. Camelids are likely to play a significant role in the epidemiology of Q fever among the human population in Pakistan and contiguous countries. Epidemiology of *C. burnetii* involves many risk factors, like age, herd size, season, sex, exposure to ticks, and bioclimatic zone while developing causal models for the disease occurrence and distribution. Coxiellosis is commonly asymptomatic; yet results in serious health problems in humans, besides reproductive issues and financial losses in animals. In brief, the presence of *C. burnetii* in dromedary camels is alarming and must be considered while developing control strategies. *C. burnetii* is a major source of infection for humans and animals.

## Data Availability Statement

The datasets presented in this study can be found in online repositories. The names of the repository/repositories and accession number(s) can be found in the article/supplementary material.

## Ethics Statement

The animal study was reviewed and approved by Blood samples were collected from camels as per the guidelines of the International Animal Care and Use Committee (IACUC) and after obtaining the written consent from the owner of the camel. The study was approved by the Directorate of Graduate Studies following the ethical guidelines of the Institutional Biosafety Committee (IBC), and the Institutional Animal Care and Use Guidelines given in the Animal Care Handbook of the University of Agriculture, Faisalabad, Pakistan (31).

## Author Contributions

SH and MS: conceptualization. SH, MT, ZA, and MG: methodology and investigation. MH: software and formal analysis. MS and MSS: validation. HE-A, KM-S, and HN: resources. SH: data curation. SH, MS, IK, and TJ: writing—original draft preparation. MS, HE-A, KM-S, MA, and HN: writing—review and editing. MS: visualization. HN and GM: supervision. MS, HE-A, KM-S, and HN: project administration. All authors read and agreed to the published version of the manuscript.

## Conflict of Interest

The authors declare that the research was conducted in the absence of any commercial or financial relationships that could be construed as a potential conflict of interest.

## Publisher's Note

All claims expressed in this article are solely those of the authors and do not necessarily represent those of their affiliated organizations, or those of the publisher, the editors and the reviewers. Any product that may be evaluated in this article, or claim that may be made by its manufacturer, is not guaranteed or endorsed by the publisher.
